# Romanian registry for interventional treatment in acute stroke: a hope for secondary prevention through medication adherence improvement

**DOI:** 10.25122/jml-2023-1015

**Published:** 2023-04

**Authors:** Cristina Tiu, Diana Alecsandra Grad, Dafin Muresanu

**Affiliations:** 1 Department of Neurology, University Emergency Hospital Bucharest, Bucharest, Romania; 2 Department of Clinical Neurosciences, Carol Davila University of Medicine and Pharmacy Bucharest, Bucharest, Romania; 3 RoNeuro Institute for Neurological Research and Diagnostic, Cluj-Napoca, Romania; 4 Department of Public Health, Babes-Bolyai University, Cluj-Napoca, Romania; 5 Department of Neurosciences, Iuliu Hatieganu University of Medicine and Pharmacy, Cluj-Napoca, Romania

Stroke is a major contributor to the global burden of disease. In 2017, stroke caused 691 age-standardized disability-adjusted life years (DALYs) per 100,000 inhabitants in Europe, second only to migraine [1]. Ischemic stroke is responsible for the highest disease burden as compared to transient ischemic attack (TIA), and hemorrhagic stroke [2]. The interplay between modifiable and non-modifiable stroke-related risk factors, encompassing environmental, genetic, and behavioral factors further underscores the need for comprehensive stroke registries [2–4]. These registries record both modifiable risk factors, such as high blood pressure, body mass index, smoking, and alcohol consumption as well as non-modifiable factors like age and sex [5,6]. However, the limited availability of stroke registries, particularly those collecting data beyond acute ischemic stroke, poses significant challenges, impacting data availability and national health services planning and delivery at all levels.

A survey reported that in early 2017 only 36.4% (out of a sample of 42) of the European countries included in the study had national stroke registries [7]. This stark disparity in registry availability across Europe hampers efforts to generate comprehensive data necessary for evidence-based decision-making and effective healthcare planning. The Action Plan for Stroke in Europe 2018-2030, drafted by the European Stroke Organisation (ESO) together with the Stroke Alliance for Europe (SAFE) highlighted the role of stroke registries in improving stroke services at various stages of the patient pathway and clinical practice [8].

According to the country health profile of Romania, 25% of all deaths were attributable to dietary risks, 17% to tobacco, 7% to alcohol consumption, and 7% to air pollution - all stroke-related risk factors which influence stroke morbidity and mortality. As for the top 10 causes of death, stroke was the second, causing 16.3% (42,569) out of the total number of deaths in 2020 [9].

Although Romania does not have a national stroke registry, the registry for interventional treatment in acute stroke (including thrombolysis and mechanical thrombectomy cases) collects data on clinical characteristics, stroke-related risk factors, and stroke outcomes. The registry also collects data on age, sex, height, weight, residency, medication usage for stroke-related risk factors, presence of other disorders, stroke quality indicators pertaining to the process of care (e.g., time from stroke onset to ambulance arrival, emergency admission, thrombolysis and mechanical thrombectomy initiation, diagnosis services), and outcomes at different timepoints following stroke onset such as admission, discharge, and at three months [10-11]. Similar variables focusing on service delivery are collected by RES-Q (the Registry of Stroke Care Quality), a multinational registry established by the European Stroke Organisation to evaluate and improve stroke care. RES-Q enrolled 614,912 patients from 2,132 sites in 91 countries. In Romania, 12,263 patients were enrolled from over 43 sites [12].

Patients have an increased risk of recurrence following an initial ischemic stroke, especially within the first months and year [13-14]. The national registry, which compiles data collected by neurology units performing thrombolysis and mechanical thrombectomies, represents a valuable resource for supporting local and public health authorities. This extensive database can be effectively used to customize programs addressing the management of identified risk factors and in secondary prevention programs focused on medication adherence in stroke patients. The registry operates under the national health program called the Priority Action for Interventional Treatment in Acute Stroke established to provide the necessary financing for intravenous thrombolysis medication, endovascular treatment of ruptured aneurysms, and instruments/kits for mechanical thrombectomy) [15].

To enhance surveillance and treatment adherence, collecting data on the medication used for managing risk factors at three months following the index stroke case (first stroke) is essential. A real-world observational study by Dalli and colleagues, drawing data from the Australian Stroke Clinical Registry and the Pharmaceutical Benefits Scheme, investigated medication (i.e., statins, antihypertensive and antithrombotic agents) adherence during the first year following an index stroke or TIA. Their findings highlighted improved survival in patients with greater adherence (especially for statins or antihypertensive agents) [16].

Furthermore, a meta-analysis on interventions designed to improve post-stroke medication adherence included 17 studies on the following interventions: dosage modification and follow-up at weeks 1 and 2; education by a clinical pharmacist at discharge; therapy initiation during inpatient and close monitoring, or educating patient at discharge; statin prescription during hospitalization; STOP (secondary stroke prevention program); PROTECT (the preventing recurrence of thromboembolic events through coordinated treatment program); educational intervention following evaluation of patient awareness; risk factor reduction (educational program), or through behavior modification; motivational interviews (counseling, regimen simplification); and environmental manipulation [17].

Evidence supports the effectiveness of interventions centered around patient counseling and education (following hospital discharge), risk factor management programs, motivational interventions targeting behavioral change, and electronic monitoring of medication adherence. For instance, in a 2-phase prospective study, Hohmann and colleagues assessed treatment adherence in ischemic stroke patients and showed that providing detailed information regarding the treatment regimen changes from hospital admission to discharge increased medication adherence in the intervention group by 8.1% (for antithrombotic drugs) and by 17.9% (for statin therapy) [18]. Menard et al. developed a systemic educational program targeting medication adherence, self-monitoring, and lifestyle modifications, improving adherence rates for antihypertensive and antiplatelet medications [19]. Sit and colleagues implemented an intervention program delivered by community registered nurses, focusing on stroke-related topics, resulting in participant improvements in self-monitoring, health literacy, and diet [20]. Similarly, a personalized intervention involving implementation intentions and identifying implementation barriers significantly increased medication adherence [21].

While implementing evidence-based interventions is crucial, data on adherence barriers are equally important for successful outcomes. For example, a qualitative study highlighted barriers faced by stroke patients, including lack of information and doubts about secondary prevention medications, stroke risk factors, and relapse possibility, as well as challenges faced by caregivers in ensuring patient medication adherence [22].

In the face of constrained health budgets and competing healthcare priorities, there is a pressing need to maximize the impact of available resources. Using the data compiled by the national thrombolysis registry for secondary preventive services, specifically by identifying medicated patients and adding several questions regarding adherence barriers, we could improve the health outcomes of individuals and mitigate the burden of stroke, and if interventions are properly implemented to reduce stroke recurrence, disability, and mortality.
